# Italians Do It … Less. COVID-19 Lockdown Impact on Sexual Activity: Evidence From a Large Representative Sample of Italian Adults

**DOI:** 10.2188/jea.JE20210055

**Published:** 2021-12-05

**Authors:** Andrea Amerio, Alessandra Lugo, Cristina Bosetti, Tiziana Fanucchi, Giuseppe Gorini, Roberta Pacifici, Anna Odone, Silvano Gallus

**Affiliations:** 1 Department of Neuroscience, Rehabilitation, Ophthalmology, Genetics, Maternal and Child Health, Section of Psychiatry, University of Genoa, Genoa, Italy; 2 Istituto di Ricovero e Cura a Carattere Scientifico IRCCS, Ospedale Policlinico San Martino, Genoa, Italy; 3 Department of Environmental Health Sciences, Istituto di Ricerche Farmacologiche Mario Negri IRCCS, Milan, Italy; 4 Department of Oncology, Istituto di Ricerche Farmacologiche Mario Negri IRCCS, Milan, Italy; 5 SOD Alcologia & Centro Alcologico Regionale Toscano, Azienda Ospedaliero-Universitaria Careggi, Florence, Italy; 6 Oncologic Network, Prevention and Research Institute ISPRO, Florence, Italy; 7 National Centre on Addiction and Doping, Istituto Superiore di Sanità, Rome, Italy; 8 Department of Public Health, Experimental and Forensic Medicine, University of Pavia, Pavia, Italy; 9 Istituto di Ricovero e Cura a Carattere Scientifico IRCCS, San Raffaele Scientific Institute, Milan, Italy

**Keywords:** COVID-19, lockdown restrictions, sexual activity

## Abstract

**Background:**

To explore how sexual activity was impacted by coronavirus disease 2019 (COVID-19) lockdown measures in the general adult population.

**Methods:**

A cross-sectional survey was conducted among 6,003 Italian adults aged 18–74 years who were representative of the Italian general population. Study subjects were recruited at the time of the nationwide stay-at-home order (from April 27 to May 3, 2020). We identified characteristics associated with decreased frequency of sex during lockdown, differentiating between cohabiting and non-cohabiting subjects.

**Results:**

Over one-third (35.3%) of Italians reported to have changed their sexual activity during lockdown (8.4% increased and 26.9% decreased). When focusing on cohabitants (*N* = 3,949, 65.8%), decreased sexual activity (20.7%) was more frequently reported by men (22.3%; compared to women, multivariable odds ratio 1.23; 95% confidence interval, 1.05–1.44), younger subjects (*P* for trend <0.001), more educated subjects (*P* for trend = 0.004), subjects living in smaller houses (*P* for trend = 0.003), and those reporting longer time spent outdoors before the lockdown (*P* for trend <0.001).

**Conclusions:**

COVID-19 lockdown drastically altered people’s day-to-day life and is likely to have impacted lifestyle habits and behavioral risk factors, including sexual attitudes and practice. This is the first national population-level study exploring changes in sexual life in this COVID-19 era. As we report sexual practice to have been affected by lockdown restrictions, we suggest that the mental health, social, and other determinants of these changes are to be explored beyond imposed social distancing.

## INTRODUCTION

Since the World Health Organization (WHO) declared the coronavirus disease 2019 (COVID-19) outbreak a pandemic on March 11^th^ 2020,^1^ rapid and severe ‘lockdown’ measures have been adopted by the Italian Government, with school closures, border restrictions, quarantine of confirmed or suspected patients, and ‘stay-at-home’ or confinement policies for all citizens.^2^

Social distancing, isolation, and infection fear drastically altered people’s day-to-day life, with higher risk of mood lability, anxiety symptoms, irritability, insomnia, and potential consequences on sexual activity and couples’ relationships.^3^ Being forced to stay together and sharing the same living space for many weeks/months could result in decreased or increased sexual desire and activity, depending on the individual response to stress.

To our knowledge, original studies investigating changes in sexual life during COVID-19 lockdown are still scant and have all been conducted on convenience samples.^4^^–^^6^ As part of the project LOckdown and lifeSTyle IN ITALY (‘LOST IN ITALY’), we conducted a large cross-sectional study during the ‘stay-at-home’ weeks to assess the impact of COVID-19 mass quarantine restrictions on lifestyle habits, social dynamics, and mental health.^7^ Within this survey, we explored reported changes in sexual activity compared to pre-lockdown times.

## METHODS

We conducted a cross-sectional study on a representative sample of 6,003 Italian adults aged 18–74 years. Study participants were recruited from April 27 to May 3, 2020 (ie, within the first phase of the lockdown, between March 9 to May 4, 2020) from the web panel of Doxa, the Italian branch of the Worldwide Independent Network/Gallup International Association, including more than 140,000 Italian adults. Using a quota sampling method by age, sex, and region (the first-level constituent Italian entity), we randomly selected 6,003 participants (2,962 men and 3,041 women) from all 140,000 panelists. The response rate was 36%. Other details of the study and sampling methodology are provided elsewhere.^8^ Recruited subjects filled an online self-administered questionnaire, including detailed information on demographic and socio-economic characteristics, such as marital status, education, number of rooms, and number of people living at home. From the number of rooms and the number of people at home, we derived the ratio of household inhabitants per room. We also collected information on selected lifestyle habits, including smoking status and time (in hours per week) spent outdoors for leisure time or work prior to lockdown. A specific question asked if participants’ sexual activity was lower, the same, or higher compared to before the lockdown. The protocol of the study was approved by the ethics committee (EC) of the coordinating group (EC of Fondazione Istituto di Ricovero e Cura a Carattere Scientifico, Istituto Neurologico Carlo Besta, File number 71-73, April 2020) and written consent to participate was collected from all study participants.

We present descriptive analyses (prevalence and 95% confidence intervals [CIs]) of overall changes, decrease, and increase in sexual activity during lockdown by sex, age group, and marital status. We derived odds ratios (OR) and corresponding 95% CIs for decreased sexual activity (ie, the most frequent outcome reported by the respondents) compared to either no change or increased sexual activity using multivariable logistic regression models, after adjustment for sex, age group, level of education, and geographic area. All estimates were provided overall and differentiating between cohabiting subjects (ie, married/cohabitants) and non-cohabiting subjects (ie, singles, divorced/separated, or widowed). A statistical weight was applied to all the analyses to further guarantee the representativeness of the national sample in terms of sex, age, socio-economic status (ie, level of education) and geographic area (ie, region and municipality size). Sampling weights were computed by Doxa using official national data for 2019 from the Italian National Institute of Statistics.

## RESULTS

**Figure 1.**
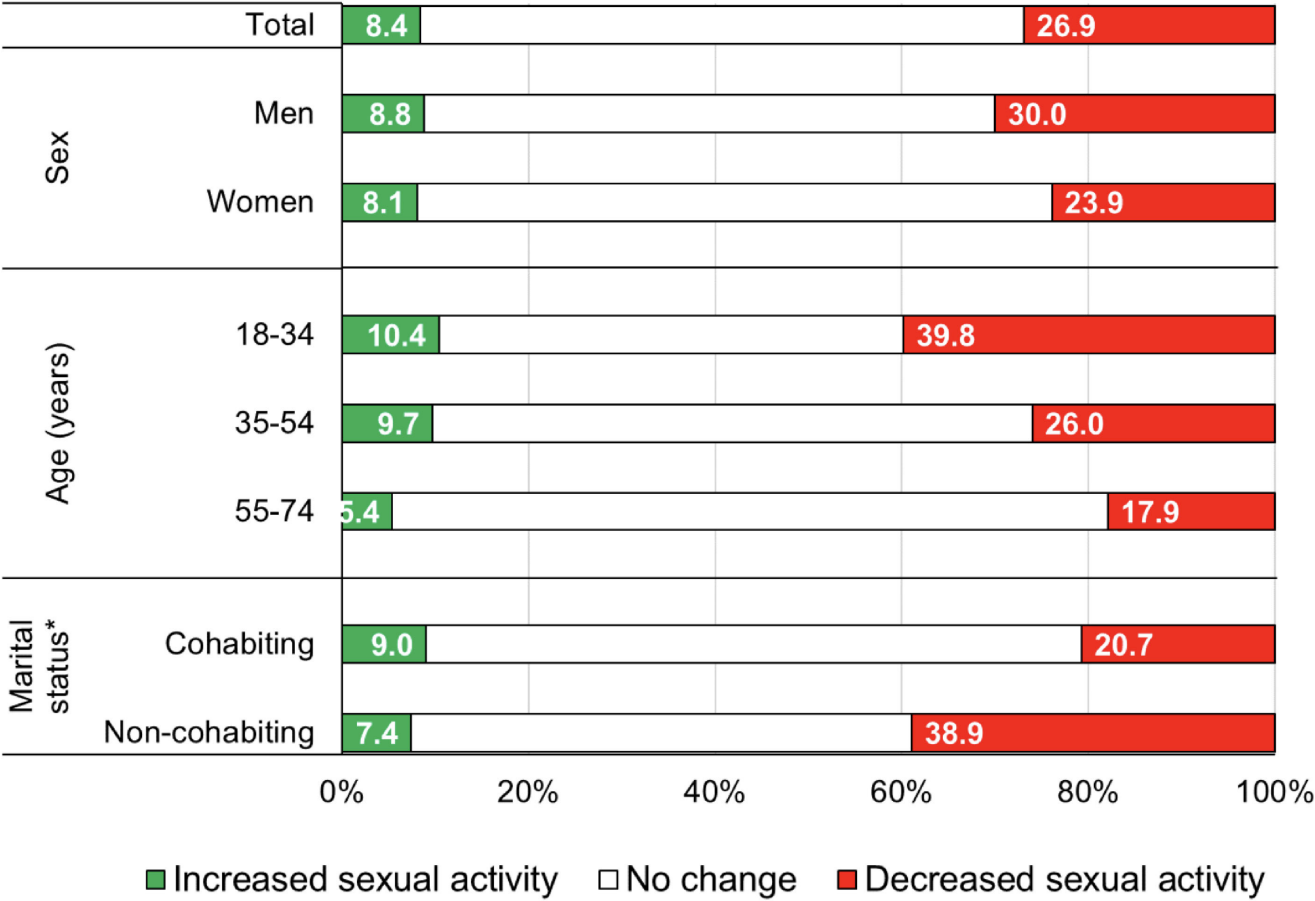
Distribution of 6,003 Italians by change in sexual activity during the COVID-19 lockdown, overall and in strata of marital status. Italy, 2020. “Cohabiting” (*N* = 3,949) includes participants reporting they were married or they were living with the partner; “Non-cohabiting” (*N* = 2,054) includes participants reporting they were divorced or separated (19.2%), widowed (5.6%), or single (75.2%). COVID-19, coronavirus disease 2019.

Among 6,003 participants, 35.3% reported to have experienced changes in sexual activity during lockdown: 8.4% reported to increase, while 26.9% reported a decrease (Figure 1). Those reporting a change (either positive or negative) in sexual activity were more frequently men (38.8%), young subjects (ie, aged 18–34; 50.2%), and singles (46.3%). By multivariable analysis, compared to cohabitants, decreased sexual activity was higher in non-cohabiting (ie, singles or divorced/separated or widowed) subjects (OR 2.06; 95% CI, 1.82–2.34; data not shown in tables).

The distribution and characteristics of Italians having decreased their sexual activity is reported in Table 1. When focusing on cohabitants (*N* = 3,949, 65.8% of total study population) decreased sexual activity was more likely reported by men (OR 1.23; 95% CI, 1.05–1.44), younger subjects (*P* for trend <0.001), more educated subjects (*P* for trend = 0.004), subjects living in smaller houses (increasing number of inhabitants per room, *P* for trend = 0.003), and those reporting longer time spent outdoors before lockdown (*P* for trend <0.001). Current smoking was associated with reported decreased sexual activity among non-cohabitants (OR 1.47; 95% CI, 1.18–1.84), but not among cohabitants.

**Table 1.** Distribution of Italians having decreased their sexual activity during the Covid-19 lockdown, according to selected demographic and socio-economic features, lifestyle habits and other individual-level characteristics, overall and in strata of marital status,^a^ Italy, 2020

	Total			Married people/cohabiting subjects			Non-cohabiting subjects (divorced/separated, widowed, single)		

	*N*	%	OR (95% CI)^b^	*N*	%	OR (95% CI)^b^	*N*	%	OR (95% CI)^b^
Total	6,003	26.9	—	3,949	20.7	—	2,054	38.9	—
Sex									
Women	3,041	23.9	1.00^c^	2,025	19.1	1.00^c^	1,015	33.6	1.00^c^
Men	2,962	30.0	**1.37 (1.22–1.54)**	1,923	22.3	**1.23 (1.05–1.44)**	1,039	44.1	**1.43 (1.19–1.72)**
Age group, years									
18–34	1,557	39.8	1.00^c^	628	22.8	1.00^c^	929	51.2	1.00^c^
35–54	2,457	26.0	**0.53 (0.47–0.61)**	1,817	22.8	0.99 (0.80–1.23)	640	35.1	**0.53 (0.43–0.66)**
55–74	1,989	17.9	**0.34 (0.29–0.39)**	1,503	17.1	**0.70 (0.56–0.89)**	485	20.3	**0.26 (0.20–0.34)**
*P* for trend			**<0.001**			**<0.001**			**<0.001**
Level of education									
Low	911	22.1	1.00^c^	612	17.4	1.00^c^	299	31.6	1.00^c^
Intermediate	3,032	27.9	**1.32 (1.10–1.58)**	1,926	19.6	1.17 (0.92–1.49)	1,106	42.4	**1.43 (1.08–1.90)**
High	2,060	27.5	**1.28 (1.06–1.55)**	1,411	23.5	**1.40 (1.09–1.78)**	649	36.4	1.23 (0.91–1.67)
*P* for trend			**0.049**			**0.004**			0.532
Number of inhabitants per room									
<1	3,429	25.0	1.00^c^	2,119	18.1	1.00^c^	1,310	36.2	1.00^c^
1	1,481	30.4	**1.18 (1.03–1.35)**	1,058	24.0	**1.40 (1.17–1.68)**	424	46.3	1.08 (0.84–1.37)
>1	1,093	28.1	1.01 (0.86–1.18)	772	23.0	**1.28 (1.04–1.58)**	321	40.3	0.80 (0.61–1.05)
*P* for trend			0.488			**0.003**			0.218
Smoking status									
Never	4,053	26.5	1.00^c^	2,626	20.6	1.00^c^	1,427	37.4	1.00^c^
Former	549	25.3	1.14 (0.92–1.41)	420	19.3	1.02 (0.78–1.33)	130	44.7	**1.95 (1.32–2.87)**
Current	1,400	28.5	**1.16 (1.01–1.34)**	903	21.3	1.04 (0.86–1.25)	498	41.7	**1.47 (1.18–1.84)**
Time spent outdoor prior to lockdown, hours/week									
0	183	13.3	1.00^c^	108	6.8	1.00^c^	75	22.7	1.00^c^
1–6	1,601	24.0	**2.04 (1.30–3.19)**	1,000	18.6	**2.97 (1.38–6.38)**	601	32.8	**1.84 (1.02–3.32)**
7–14	2,139	24.7	**2.29 (1.47–3.58)**	1,444	20.1	**3.36 (1.57–7.17)**	696	34.3	**2.07 (1.16–3.72)**
≥15	2,079	32.6	**3.36 (2.16–5.24)**	1,396	23.7	**4.26 (1.99–9.10)**	683	50.8	**3.89 (2.17–6.97)**
*P* for trend			**<0.001**			**<0.001**			**<0.001**

CI, confidence interval; OR, odds ratio.

^a^Participants were classified on the basis of their marital status in married people/cohabiting subjects (ie, participants answering they were married or they were living with the partner) and in non-cohabiting subjects (ie, participants answering they were divorced or separated, widowed or single).

^b^Estimated by multivariable logistic regression models after adjustment for sex, age group (18–34, 35–54, 55–74), level of education (low: up to middle school diploma, intermediate: high school, high: university) and geographic area (Northern Italy, Central Italy, Southern Italy and islands). Statistically significant estimates at 0.05 level are in bold.

^c^Reference category.

Analyses for cohabitants stratified by sex are shown in eTable 1. Results for age, smoking status, and time spent outdoors were consistent in both sexes. More educated men (*P* for trend <0.001) and women living in smaller houses (*P* for trend = 0.001) significantly decreased sexual activity.

Among cohabitants, increased sexual activity was more frequently reported by men (OR 1.43; 95% CI, 1.14–1.79), young (*P* for trend <0.001), and subjects living in larger houses (*P* for trend = 0.023; data not shown in tables). No relationship has been observed with level of education, smoking status, and time spent outside.

## DISCUSSION

We report that more than one-third of Italian adults modified their frequency of sex under COVID-19 lockdown, with one in four Italians reporting a decrease. While decreased sexual activity during the nationwide stay-at-home order was expected in singles or non-cohabitants, one in five married or cohabiting subjects reporting decreased sexual activity is something worth exploring. We estimate that, within cohabitants, men, younger generations, more educated subjects, people living in smaller houses, and those who used to spend more time outdoors prior to lockdown were more likely to report decreased sexual activity. Men and the young also reported an increase in their sexual activity. These subgroups of the population were more susceptible to any change (either increase or decrease) in sexual activity.

Several environmental factors, including behavioral and psychological determinants, are known to be associated with sexual life^9^; however, as most of the available evidence on the topic is derived from cross-sectional studies, their causative effect is far from being established. In the context of the COVID-19 outbreak, sexual practice and attitudes might have been influenced through different pathways.^10^ These include: i) modified opportunities for sexual intercourses (imposed social distancing and isolation rules may have prevented people from meeting [mainly singles and non-cohabitants] or, alternatively, they may have forced cohabiting subjects [mainly married/cohabitants] to spend long hours together); ii) fear of infection transmission during close contacts; and iii) psychological stress associated with the COVID-19 public health emergency and its clinical, social, and economic consequences, as mediators of sexual desire. These potential mechanisms might explain the large proportion of Italian adults having modified (mostly decreased) their sexual activity.

In fact, if we apply these theoretical pathways to interpret our data, we can confirm that limiting the opportunity for casual sexual encounters as a consequence of imposed social contact restrictions reduced reported sex frequency in singles and young people. On the other hand, even if married/cohabitants might consider intimacy as a way to enhance their relationship during long hours spent at home, feelings of apprehension and anxiety might have prevailed, ultimately making sex not desirable or enjoyable.^11^ Reported sex differences in married/cohabitants might be partly due to men stopping extramarital sexual relations—including with sex workers—during lockdown.^12^ In addition, information bias cannot be ruled out: as men have shown to overestimate the number of reported sexual intercourses and partners compared to women,^13^ such tendency might having played in the opposite direction when overestimating having to renounce sex due to COVID-19.

The association between decreased sex frequency in people living in smaller houses can be interpreted both in light of lack of privacy to have sex, as well as a proxy for lower socio-economic status, with consequent higher risk of anxiety associated to household-level economic impact of COVID-19 response and decrease willingness to enjoy sex.^14^

Findings from our survey suggest decreased sexual activity in more educated subjects who might have adopted a more cautious approach to sex being more aware of the public health emergency and associated risks.^15^

Subjects reporting longer time spent outdoors before the lockdown might be more likely to have more unstable relationships or to have suffered more from staying at home, with ultimate consequences on sexual desire.^16^

Smoking is a well-known risk factor of sexual dysfunction that could impact the quality of life by reducing sexual desire and satisfaction and damaging self-esteem and interpersonal relationship.^17^ A positive dose-dependent association between smoking and both male erectile dysfunction and female sexual dysfunction in smokers has been extensively studied.^18^^,^^19^ In light of the increase in smoking reported during lockdown,^7^ cigarette smoking might have had a role in the observed decreased sexual activity among smokers.

Pre-COVID-19 population-level surveys on patterns and characteristic of sexual activity are available for selected countries, including the United Kingdom,^20^ the United States,^21^ Scandinavian countries,^22^ Australia,^23^ and Japan,^24^ among others, showing that they are setting-specific and reflect cultural trends. The public health relevance of exploring sexual habits and attitudes has been previously recognised.^25^ From a public health perspective, it is important to identify both determinants of sexual activity and the effects of sexual activity on mental and physical health. As the COVID-19 public health emergency and response is projected to have deep health, social, and economic impacts,^26^^,^^27^ we believe it is worth exploring how it has impacted sexual and reproductive health and their associated factors.

Our findings, although preliminary and with limitations linked to the cross-sectional study design, provide timely estimates of the lockdown’s impact on sexual activity, finding that more than one-third of the population reported sexual life to be affected by lockdown, and suggest that the mental health, social, and other determinants of these changes are to be explored beyond imposed social distancing. Almost no evidence exists on the topic; one cross-sectional study conducted in Bangladesh, India, and Nepal reported the percentage of married couples having sexual intercourse more than five times per week to have increased from 6.7% to 10% before and during COVID-19 lockdown; however, no reliable conclusions can be derived from such data, as the survey was conducted on a convenience sample of 120 subjects.^4^

To our knowledge, this is the first study on the topic conducted in a large national representative sample. As it is part of a broader study exploring the effects of the COVID-19 public health response in Italy on a rich set of behavioral risk factors and physical and mental health outcomes through both cross-sectional and longitudinal study designs, we are confident the current data will help to better design complementary research questions in the near future, so as to provide solid and comprehensive national-level evidence that can inform the planning, implementation, and monitoring of mitigation interventions of the adverse health and social consequences of the current pandemic.

## APPENDIX A. SUPPLEMENTARY DATA

eTable 1. Distribution of married/cohabiting Italian subjects having decreased their sexual activity during the COVID-19 lockdown, according to selected demographic and socio-economic features, lifestyle habits and other individual-level characteristics, in strata of sex, Italy, 2020^a^
