# Supplementary material for: Italians Do It … Less. COVID-19 Lockdown Impact on Sexual Activity: Evidence From a Large Representative Sample of Italian Adults
Source: J Epidemiol. 2021 Dec 5;31(12):648–52. doi: 10.2188/jea.JE20210055 (PMC8593579; doi:10.2188/jea.JE20210055)
Supplement: Supplementary file 1 [file je-31-648-s001.pdf]

eTable 1. Distribution of married/cohabiting Italian subjects having decreased their sexual activity during the COVID-19 lockdown, according to selected demographic and socio-economic features, lifestyle habits and other individual-level characteristics, in strata of sex, Italy, 2020[a]

	**Men**			**Women**		
	**N**	**%**	**OR (95% CI)**	**N**	**%**	**OR (95% CI)**
Total	1,923	22.3	-	2,025	19.1	-
Age group, years						
18–34	277	24.4	1.00[b]	350	21.5	1.00[b]
35–54	894	25.0	1.05 (0.77–1.44)	923	20.7	0.94 (0.70–1.27)
55–74	751	18.3	0.73 (0.52–1.02)	752	15.9	**0.68 (0.49–0.94)**
p for trend			**0.012**			**0.008**
Level of education						
Low	371	16.2	1.00[b]	241	19.3	1.00[b]
Intermediate	816	20.9	1.36 (0.98–1.89)	1,110	18.7	0.91 (0.64–1.30)
High	737	27.0	**1.83 (1.33–2.53)**	674	19.6	0.94 (0.64–1.37)
p for trend			**<0.001**			0.893
Number of inhabitants per room						
<1	1,025	19.8	1.00[b]	1,094	16.5	1.00[b]
1	513	26.4	**1.43 (1.11–1.85)**	544	21.8	**1.38 (1.06–1.80)**
>1	385	23.5	1.19 (0.89–1.59)	387	22.6	**1.39 (1.04–1.87)**
p for trend			0.087			**0.011**
Smoking status						
Never	1,207	22.5	1.00[b]	1,420	19.0	1.00[b]
Former	253	22.1	1.23 (0.88–1.73)	167	15.1	0.78 (0.50–1.22)
Current	463	21.9	1.01 (0.78–1.31)	439	20.7	1.09 (0.83–1.42)
Time spent outdoor prior to lockdown (hours/week)						
0	41	5.6	1.00[b]	67	7.6	1.00[b]
1–6	433	18.1	3.57 (0.92–13.9)	567	19.0	**2.81 (1.11–7.11)**
7–14	638	21.8	**4.68 (1.22–18.0)**	806	18.8	**2.86 (1.14–7.20)**
≥15	811	25.8	**6.43 (1.68–24.7)**	585	20.8	**3.30 (1.30–8.32)**
p for trend			**<0.001**			**0.041**

CI, confidence interval; COVID-19, coronavirus disease 2019; OR, odds ratio.

[a] Estimated using multiple logistic regression models after adjustment for age group, level of education and geographic area. Statistically significant estimates at 0.05 level are in bold.

[b] Reference category.
